# Investigation into combination of an antimicrobial peptide with existing antibiotics against antibiotic resistant clinical isolates of Escherichia coli

**DOI:** 10.1186/2047-2994-4-S1-I5

**Published:** 2015-06-16

**Authors:** Y Hu, J Shallop, Y Liu, A Coates

**Affiliations:** 1 Infection and Immunity, St George's University of London, UK

## Introduction

Bacterial infections remain the leading killer worldwide which is worsened by the continuous emergence of antibiotic resistance. In particular, antibiotic resistant Gram-negative bacteria are prevalent and extremely difficult to treat. Therefore, rejuvenating the therapeutic potentials of existing antibiotics represents an attractive novel strategy. Antimicrobial peptides have been of great focus recently. Newly derived synthetic lipopeptides have been shown to exhibit antimicrobial activity against bacteria and fungi.

## Objectives

In this study, we investigated the ability of an antimicrobial peptide, PA-KKkK, to enhance the potency of currently used antibiotics against antibiotic-resistant clinical isolates of *Escherichia coli* and a NDM-1 producing strain.

## Methods

Antibiotic susceptibility was investigated by determining the minimal inhibitory concentration (MIC) using a broth dilution method. To study the combined interactions between PA-KKkK and the antibiotics, chequerboard titrations were performed. Time-kill assays were then carried out to prove the effect of synergistic activity against the tested bacterial strains. Transmembrane potential depolarisation assays and ATP levels were determined to understand the mechanism of action of PA-KKkK.

## Results

NDM-1 producing strain was extremely resistant to all antibiotics tested. The factional inhibitory concentration index (FICI) was calculated to show the peptide synergised with rifampicin against the NDM-1 strain, while it synergised with rifampicin, colistin, ceftazidime and aztreonam against antibiotic-resistant clinical isolate of *E. coli*. Time-kill analysis demonstrated significant synergistic activities when a low level of PA-KKkK was combined with rifampicin and colistin. PA-KKkK had a membranolytic effect on cytoplasmic membrane and in combination, decreased ATP levels of cells in a dose-dependent manner.

## Conclusion

We have demonstrated that PA-KKkK acts as an antibiotic enhancer and therefore accelerates the bactericidal activity of drugs against antibiotic-resistant *E. coli*. This novel treatment regimen can have major clinical implications in our fight against Gram-negative bacterial infections.

## Disclosure of interest

None declared.

